# Factors influencing the use of topical repellents: implications for the effectiveness of malaria elimination strategies

**DOI:** 10.1038/srep16847

**Published:** 2015-11-17

**Authors:** Charlotte Gryseels, Sambunny Uk, Vincent Sluydts, Lies Durnez, Pisen Phoeuk, Sokha Suon, Srun Set, Somony Heng, Sovannaroth Siv, René Gerrets, Sochantha Tho, Marc Coosemans, Koen Peeters Grietens

**Affiliations:** 1 Institute of Tropical Medicine, Antwerp, Belgium; 2 Amsterdam Institute of Social Science Research, University of Amsterdam, The Netherlands; 3 National Center for Parasitology, Entomology and Malaria Control, Phnom Penh, Cambodia; 4 University of Antwerp, Antwerp, Belgium; 5 Partners for Applied Social Sciences (PASS) International, Tessenderlo, Belgium; 6 School of International Health Development, Nagasaki University, Nagasaki, Japan

## Abstract

In Cambodia, despite an impressive decline in prevalence over the last 10 years, malaria is still a public health problem in some parts of the country. This is partly due to vectors that bite early and outdoors reducing the effectiveness of measures such as Long-Lasting Insecticidal Nets. Repellents have been suggested as an additional control measure in such settings. As part of a cluster-randomized trial on the effectiveness of topical repellents in controlling malaria infections at community level, a mixed-methods study assessed user rates and determinants of use. Repellents were made widely available and Picaridin repellent reduced 97% of mosquito bites. However, despite high acceptability, daily use was observed to be low (8%) and did not correspond to the reported use in surveys (around 70%). The levels of use aimed for by the trial were never reached as the population used it variably across place (forest, farms and villages) and time (seasons), or in alternative applications (spraying on insects, on bed nets, etc.). These findings show the key role of human behavior in the effectiveness of malaria preventive measures, questioning whether malaria in low endemic settings can be reduced substantially by introducing measures without researching and optimizing community involvement strategies.

Although parasites, vectors and humans all play a key role in malaria transmission, human behaviour in all its diversity and variability is not always sufficiently considered in prevention policies^1^,^2^,^3^,^4^. This is reflected in the unprecedented decrease in malaria in the Greater Mekong Sub-region over the last 10 years in general but not in minority populations, such as the indigenous people of the Cambodian province Ratanakiri. The province is mainly populated by ethnic minorities, socio-culturally and linguistically different from the majority Khmer population of Cambodia^5^,^6^,^7^,^8^. In terms of the mosquito population, local vectors have been shown to bite early and outdoors. Even with an optimal coverage of Long Lasting Insecticidal Nets (LLIN) or Indoor Residual Spraying (IRS), malaria transmission may still continue as vectors escape contact with insecticide treated surfaces while their blood meals are still active outdoors^9^,^10^,^11^. The progressive confinement of malaria to specific risk populations such as ethnic minorities and forest workers, and the subsequent complex interplay between human and mosquito behaviour^9^, calls for innovative measures adapted to local circumstances^9^,^12^,^13^.

Several additional measures to LLINs have been suggested in light of current elimination goals, such as toxic sugar baits which attract and kill mosquitoes^14^, insecticide-treated clothing^15^,^16^, insecticide-treated hammock nets^2^,^17^ and spatial repellents^18^,^19^. To fill the gap in the evenings and mornings when people are still active outdoors, topical repellents have also been suggested as a potentially useful measure for malaria elimination^11^,^20^. Repellents have been shown to offer personal protection against mosquito bites^21^,^22^,^23^,^24^, as a stand-alone measure^25^,^26^ or in combination with LLINs^27^. However, topical repellents require daily application by the study population in order to be effective, which is often cited to be a major challenge in repellent interventions^25^,^28^,^29^,^30^.

In Cambodia, a cluster randomized epidemiological trial was recently conducted to raise evidence on the effectiveness of the mass use of topical repellents at community level in addition to the use of LLINs in controlling malaria infections (hereafter referred to as MalaResT). In contrast to the personal protection envisioned by previous repellent studies, the MalaResT study aimed for *community* protection, meaning that diversion of mosquitos from users to non-users is avoided by the expected effect on vector populations of large-scale effective repellent use^28^. The trial was conducted from April 2012 to December 2013, and consisted of a control arm where LLIN were distributed by the National Malaria Control Program (NMCP), and an intervention arm where in addition to LLINs topical repellents were distributed. The topical repellent used in this study was Picaridin (KBR3023), which is safe^31^ and effective against local vector species^23^. A lotion formulation of 10% was used for children between 2 and 10 years old and a spray formulation of 20% from 11 years old onward. Here results are presented from the anthropological study conducted within but independent of the epidemiological trial, of which the primary objective was to acquire an in-depth understanding of the factors influencing the use or non-use of the distributed repellents. In this paper only results directly related to the use of repellents are presented. Other results stemming from this study are reported elsewhere (1,9,32,33).

## Methodology

### Study site and population

#### Study site

The study took place in the malaria endemic province of Ratanakiri in Northeastern Cambodia. The border province is geographically and politically located at the fringes of the nation-state and represents one of the least developed provinces in the country. In remote villages of Ratanakiri, Village Malaria Workers (VMW) are trained to diagnose with rapid diagnostic tests and treat positive malaria cases with antimalarials^1^,^34^. The pluralistic medical system in Ratanakiri is composed of public sector health facilities with free-of-charge combination therapies; private pharmacies selling “cocktails”, artemether injections and subsidized combination therapies; and local diviners prescribing animal sacrifices to appease the spirits^1^. LLINs are distributed free of charge at 1 net per 1 person by the NMCP and are currently the main malaria prevention tool in the study area.

#### Study population

The local population is almost exclusively composed of the following ethnic groups: Jarai, Tompuon, Kreung, Prov, Kachok, Kavet, Lon, Lao and Cham, although more recently an influx of lowland Khmer looking for economic opportunities has been observed^35^. Among these ethnic minority groups, the main revenue is generated by subsistence slash-and-burn farming on plots located near or in the forest and, less frequently, on wet rice fields. People move to live on their farms and fields in the rainy season when the workload is heaviest, coinciding with the malaria peak season. Forest farming exposes them to malaria due to the sylvatic nature of *Anopheles dirus*, the main malaria vector of the region, especially when staying overnight in homes at their farms for extended periods during the rainy season^1^,^9^,^32^,^36^,^37^. Even with the current optimal LLIN distribution, effective *use* of LLINs in villages and at farms remains suboptimal and as such constitutes a major bottleneck for effective malaria control^9^,^33^.

#### Malaria

Malaria transmission is perennial with two peaks, June-July and October-November, the rainy season lasting from May to October. At the end of the malaria season of 2012, the overall PCR prevalence in Ratanakiri, as recorded by the MalaResT study, was estimated at 4.9%^38^. Sleeping overnight at plot huts at the farm has been identified as a risk factor for malaria^32^.

### Study design

**Figure 1:**
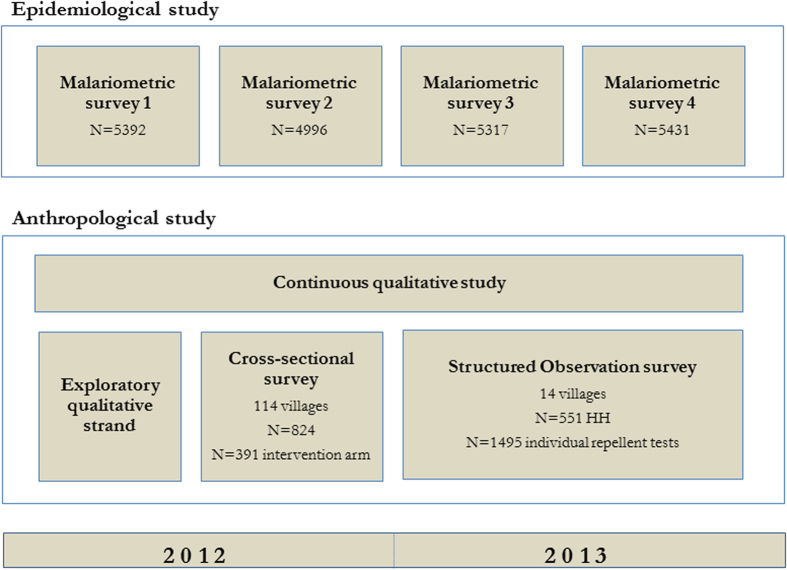
Flow-chart of the methodology used in the (i) epidemiological trial and (ii) the concurrent anthropological study.

An anthropological study investigating the acceptance of and adherence to topical repellents and existing measures such as LLINs was part of the MalaResT trial (Fig. 1). The anthropological study consisted of a mixed-methods design, combining qualitative and quantitative research methods. Such a design allows the combination of self-reporting data collection (surveys and interviews) and respondent-independent data (participant observation), limiting the expected reporting bias to questions related to the adherence of public health interventions. During the initial phase of the research in 2012, qualitative data gathering was prioritized to gain an in-depth understanding of those factors that influence people’s acceptance of and adherence to topical repellents and control measures in general. Based on the qualitative data, a cross-sectional survey using a structured closed questionnaire was designed and conducted from August until November 2012. Due to the expected high response bias, a quantitative structured observation survey was conducted from May through December 2013. Additionally, a short questionnaire was done with individuals participating in the biyearly cross-sectional malariometric surveys designed to assess the additional effect of widespread repellent use on malaria prevalence.

### Qualitative study

#### Data collection

Fieldwork, continuously conducted throughout 2012 and 2013 in ethnic minority villages in the districts of Voen Sai, Oyadao, Borkeo and Lumphat, consisted of participant observation and face-to-face open-ended interviews and informal conversations, guided by an continuously adapted interview guide in line with an emergent theory study design. All interviews, conversations and observations were conducted by the first author (CG) (ITM, Antwerp), 1 Khmer female (US) and 3 Khmer male social scientists (PP, SS and SS) (CNM, Phnom Penh). Sampling of informants was theoretical (i.e. purposively selected based on emerging results). Participants were selected based on criteria such as gender, age, social position, reported repellent access and use, professional and economic strategies (including agricultural and forest activities) and were always approached face-to-face for social interaction. Access to respondents was often granted through snowball-sampling techniques, where certain key-informants introduce the researcher to other potential participants. Many informants were visited several times as an additional way of building confidence between researcher and respondent. A total of 320 interviews were audio-recorded and transcribed, including both short informal conversations and more in-depth individual interviews. Participant observation consisted of the research team actively participating in the everyday life of the study population and observing the study setting in its day-to-day and night-time context, including overnight stays in the study villages and observation sessions at the district health centers where repellent distributor meetings were held. Continuous unrecorded informal conversations with respondents built up the confidence needed to discuss more sensitive issues such as adherence to public health interventions; 759 such informal conversations were carried out and recorded in field notes taken immediately after. Additional reflexive field notes were kept throughout the research process and included in the analysis in the form of memos.

#### Data analysis

Data analysis was concurrent to data collection. In the initial exploratory phase, inductive or open coding of raw data was preferred. When preliminary results started emerging, new hypotheses and theories were formed and tested in the field until saturation was reached. Axial coding was performed to facilitate the analytic process. NVivo 9 Qualitative Analysis Software was used for all data management and analysis.

### Quantitative study

#### Data collection

Based on preliminary results from the qualitative strand in 2012, quantitative data were systematically gathered using two surveys.

Cross-sectional survey. First a cross-sectional survey was carried out focusing on the multiple residence system, repellent use, net ownership and use, evening social activities, use of malaria preventive measures other than nets, mosquito nuisance and malaria treatment. For this survey, 450 individuals from the intervention arm of the epidemiological trial were randomly selected from the census list. A total of 393 people from 56 different intervention villages answered the questionnaire. Of the 57 individuals that were selected but not included in the survey, two were refusals; all other cases were individuals (i) whose names did not exist in the village although they were listed in the census, (ii) were mentally or physically not able to respond due to severe illness, (iii) had moved to another village or province, (iv) could not be located after three visits.

Structured observation survey. In a second quantitative phase, a structured observation survey was carried out. Households were visited unannounced in the evening between 18:30 and 20:30, when according to the guidelines of the trial the repellent should have been applied. The aim was to establish repellent use of all household members through participant observation techniques, administer a short questionnaire on repellent use, and record characteristics of bed nets in the household. The following morning the same household was visited again by the same interviewer for the second part of the structured questionnaire with the household leader, mainly focusing on socio-economic status, seasonal sleeping spaces, (alternative) use of nets, (alternative) repellent use, mosquito nuisance, and previous malaria episodes. For this survey, 10 intervention villages were selected with good access to repellents. In each village, half of all households were randomly selected from the population census. A rotation system of villages allowed for most of the villages to be visited throughout the rainy season from May until December to account for potential seasonal variation across the data. Based on previous qualitative research, it was known that the majority of households had a farm (and/or rice field) and commuted between farm- and village house, favoring the farmhouse during the rainy season and the village house during the dry season, and that this was a malaria risk factor^32^. Each selected household was thus assigned to either a ‘farm list’ (i.e. to be observed and interviewed on farm or rice field) or to a ‘village list’ (i.e. to be observed and interviewed in their village house) to explore potential differences between locations. A total of 517 households were randomly selected for the farm lists, of which 392 households were eligible (i.e. stayed overnight at farm); and 221 were reached. A total of 519 households were randomly selected for the village lists, of which 400 were eligible (i.e. stayed overnight in the village); and 210 were reached. The main reason for not reaching households was because the selected family was not staying overnight at the respective location within the timeframe of the interviewer’s stay in the village. Finally, a total of 431 intervention households were observed and interviewed, corresponding to 1495 individuals of whom repellent use was assessed.

The observational technique used consisted of an interviewer spending the evening in a randomly selected household, and -after individual consent- asking permission to smell each household member’s arm for traces of repellent in relation to the smell of the repellent. The qualitative strand indicated that perceptions regarding smell were a key factor for use. This approach was used in order to limit response bias and was preferred over directly observing the repellent being used, as the observer’s presence was expected to directly influence the decision to use the repellent (often described as the Hawthorne effect, leading to social desirability bias^39^). During the observation, participants who had not used the repellent, were expected to say so when the interviewer tried to smell their arm.

##### Malariometric survey

In the epidemiological study, malariometric surveys were performed twice a year (baseline at the start of the rainy season and follow-up towards the end), and consisted of a blood prick alongside a short questionnaire assessing net- and repellent use yesterday and last week. For each of the malariometric surveys, 65 individuals per cluster were sampled randomly (Fig. 1). An additional list of 15 randomly sampled individuals was used for those clusters where initial response was lacking. Only results from the two follow-up surveys in the intervention arm are shown here.

#### Data analysis

Preliminary analysis of the qualitative data was used to build the standardized questionnaires used in both quantitative surveys. Quantitative data were entered in Epi Info 7 and analyzed in SPSS (IBM SPSS Statistics 19). Descriptive statistics were performed and significance of relationships between variables tested using chi^2^-tests. Multivariate analysis tested the effect of age, sex, village, location of interview and month of interview on repellent use, of which only age was significant. The variation in observed repellent used between households was explored in an empty random effects model with only household entered as an intercept.

### Ethical Clearance

The study protocol, including the anthropological work package, was approved by the National Ethics Committee for Health Research in Cambodia, the Ethics Committee of the University Hospital of Antwerp, and the Institutional Review Board of the Institute of Tropical Medicine of Antwerp. For the anthropological part, the interviewers followed the Code of Ethics of the American Anthropological Association (AAA). Informed consent was obtained from all research participants.

## Results

### Acceptability

#### Conceptualization of the repellent

More than half of respondents perceived the repellent to be some type of medicine and a similar number conceived the repellent to be some type of poison (Table 1). Qualitative data related these conceptualizations to the strong smell of the repellent. Although the perceived toxicity of the product raised some concerns in the study population, especially for its application on children, it was also considered a required characteristic of the repellent in order to be effective. As such, 37.2% considered the repellent as being both a poison and a medicine.

#### Perceived benefits of the repellent

Effectiveness. Almost all respondents reported mosquitos and other insects to stop biting after spraying the repellent. More than half of respondents believed that in the second year of the trial, mosquito densities were reduced because of the repellent (Table 1).

Alternative uses. A high level of reported acceptance of the repellent was predominantly due to the non-prescribed usages of the repellent. The majority of household leaders reported to spray the repellent directly on insects (77.3%), in the air around the body (70.8%), on the outside of the bed net (62.6%), on their clothes (57.5%), on the walls of the house (53.1%), and on the inside of the bed net (45.7%) (see Table 1 for more details). Additional alternative uses that were not quantified included its use against hair lice, forest leeches and maggots in animal wounds.

#### Perceived inconveniences of the repellent

Most respondents stated to have experienced skin-related inconveniences (itchy skin, hot skin, skin rash, dry skin) because of the repellent (86.0%). About a third of respondents complained about getting flu-like symptoms and about a fifth of respondents claimed to get dizzy, to get a headache or even to feel like vomiting when spraying repellent (Table 1).

### Use

#### Use at multiple residences

##### Accessibility

Although the qualitative study indicated the local repellent distributors experienced difficulties and reluctance to travel to the farms where many villagers reside in the rainy season, in the first year of the trial almost all respondents reported having received repellents the month prior to the survey (Table 2). A large majority (81.7%) considered the amount of repellent distributed enough, although 36.9% of respondents mentioned having run out before the next encounter with the distributor.

##### Reported use

(i) During the cross-sectional survey, 73.3% reported to have used the repellent the day prior to the interview (Table 2). Reported daily evening use when checking for each location of residence separately in the same questionnaire was lower (56.5% in village, 54.6% at the farm, 51.0% at rice field). Only 34.1% of respondents answered to use the repellent across the variety of repellent use questions throughout the questionnaire (i.e. yesterday evening, this morning, always, 7 days a week). (ii) Reported daily use during the structured observation survey similarly resulted in a daily use of around 50% across the different locations (Table 2). (iii) According to the malariometric surveys of year 1 and 2, around 70% of respondents reported to have used the repellent the day prior to the survey (Table 3).

In both intervention years, around 75% of those household leaders that regularly go to the deep forest, reported to always use the repellent there, with a median of 3 applications per day (Table 2).

When cross-checking quantitative results in the qualitative study, indeed a strong preference among men for using the repellent could be observed, especially while performing deep forest activities such as hunting, logging and fishing. Women and children used repellents only sporadically, especially when residing in the village.

##### Observed use

The observational study, aiming to confirm and refute preliminary results both from the qualitative study and the cross-sectional survey, showed that 7.9% of participants had used the repellent the evening of the visit (Table 4). Among those participants that were observed in the village, 7.1% had used the repellent; among those observed at the farm, 5.9% and at rice fields 15.4%. Age was significantly associated with repellent use: children under 11 years old who use the lotion formulation of the repellent had used it significantly more often than those using the spray formulation of the repellent (Table 4). Of those respondents that had not used the repellent on the evening the survey took place, 87.1% reported to still occasionally use the repellent; 12.9% said to never use the repellent. Although usually not all members of one household had used the repellent (except for households that consisted of only husband and wife), repellent use was significantly clustered by household. The variation between households explained 40% of the total variance (data not shown in tables).

##### Reported vs. observed use

Among village respondents who reported using the repellent 7 days a week while in the village, only 10.1% were observed to have used the repellent on the evening of the interview (Table 5). Among respondents observed and interviewed at the farm, observed use among those who reported maximal adherence to the repellent when at the farm was 7.9%; at the rice field, observed use was 18.2% among those who reported maximal adherence at this location.

#### Reasons for use or non-use

##### Use

In the cross-sectional survey, the large majority of repellent users (75.1%) reported to use the repellent in order to protect themselves from mosquito bites, less so for protection from malaria (28.8%) (Table 6). Although protection from malaria was not reported to be an important reason for using the repellent, 83.0% of respondents stated that they themselves, or one of the household members, had had malaria before. During the structured observation survey, 45.4% reported they or a household member had had malaria in the last year. Substantially more people used the repellent during those months of heavy rain (August, September, October) (data not shown) when almost all respondents perceived greater mosquito- and insect nuisance as compared to drier months (Table 7). Perceived nuisance (from insects but also leeches), moreover, was highest in the forest, corresponding to the high reported repellent use among those working in the forest (74%) (Table 2).

##### Non-use

About half of respondents that had not used the repellent during the visit reported to use the repellent later in the evening. Other reported reasons were having run out of repellent, forgetting to use the repellent, not liking the smell of the repellent or a fear of side-effects (Table 6). The 12.9% that reported to never use any repellent, did so mainly because of the smell and a fear or previous experience of adverse effects (data not shown in tables).

According to the qualitative strand, the strong smell and the perceived toxicity were the main reasons for women not using the repellent on themselves and their children. This was especially the case for pregnant women who are considered generally more sensitive to strong smells and who were worried about the effects on the pregnancy. Parents also reported to be too busy in the evening with cooking and other household chores to apply the repellent to small children. Observations indicated that most people simply ‘forgot’ to use the repellent as it was not part of their established daily routine.

#### Repellent use in relation to other preventive measures

The qualitative study showed that people use smoke for warding off mosquitos while they are still active outside. Among those who use repellents and make fires, 14.4% report to no longer use the repellent when already using smoke from fire. Among repellent users and smokers, 21.5% reported no longer using the repellent when smoking cigarettes (Table 6). When looking only at those who use both repellents and bed nets, 15.2% reported to use their bed nets less often when using the repellent. Qualitative data indicated that the repellent could provide the comfort needed to sleep without a bed net during particularly hot nights, when bed nets are taken out for washing, and when bed nets tear beyond repair and new ones are not yet purchased from the market.

## Discussion

The trial study population was expected to use topical repellents on a daily basis with the aim of maximizing the community-wide protective potential of repellents. Access to repellents was assured^40^ and acceptance of the product high. Moreover, entomological data show that the Picaridin repellent reduces 97% of mosquito bites during five hours in similar settings and this without declining efficacy over time^23^. However, no reduction in malaria prevalence could be recorded at the end of the cluster-randomized trial (M. Coosemans, personal communication), suggesting that the effectiveness of the intervention mainly depended on human behavior, possibly in combination with potential effects of the repellent on vector behavior. Both a systematic review of repellent interventions^30^ and mathematical modelling^28^ have shown that “user compliance” is indeed one of the most decisive factors for the success of such an intervention. In the MalaResT trial, reported *use yesterday* was 73%, and reported *daily use* about 34%. In contrast, observed daily use was estimated at 8%, far below the minimum required coverage to obtain a mass effect on the vector population and thus on malaria transmission and prevalence^28^. Limited use of repellents was also reported in other repellent studies. One of the study limitations of a repellent intervention in Tanzania^29^ was the difficulty of achieving daily repellent application by all household members. Similarly, in a repellent study in Afghanistan, no significant reduction of malaria infections could be shown in adults over 20 years old, presumably because of reluctance among adults to adapt to using the new product daily^25^.

### Measuring use

In addition to the difficulties involved in achieving a high uptake, measuring “user compliance” is in itself an issue that is not often scrutinized. Assessing ‘use’ is complex, partly due to the response bias inherent in self-reporting methods for public health interventions^1^,^2^,^3^,^41^. In a repellent trial in the Bolivian Amazon^26^, self-reporting was recognized to be an unreliable measure of use and this was consequently measured by combining results from monthly questionnaires; records on the liquid left in bottles; and, unannounced evening “sniff checks”. In both Tanzania and Laos^29^,^41^, reported “user compliance” was compared to the amount of repellent liquid used in bottles. In Laos, where “compliance” was measured by comparing self-reported use with the amount of repellent left in returned bottles, this resulted in a 40% bias or “false positive rate”^41^. In this study, an emergent theory mixed-methods design was used in order to target difficulties in measuring and understanding ‘use’. This was done by relating data from qualitative and quantitative methods for triangulation and complementarity purposes. Structured observation, less sensitive to social desirability bias, measured a considerably lower daily use than self-reported use recorded in surveys. Although logistically a difficult undertaking, such a respondent-independent technique proved a more suitable method for estimating use as it showed an 82 to 92% difference between people reporting to use the repellent 7 days a week and those observed to use the repellent the evening of the visit in the same location. Farm-respondents’ response on whether they used repellents when sleeping in the village was 7 times higher than the observed use among farmers staying in the village, showing that the level of biased response was likely not overestimated. Moreover, as shown by the myriad of alternative uses of the repellent, the repellent’s purpose was conceptually redefined by the end users, increasing the difficulty of recording use without qualitative research informing the quantitative surveys. In public health research, the use of malaria preventive tools is often measured by individual indicators that can only detect a homogeneous and constant use while considerable accuracy could be gained from the triangulation with other methods/disciplines, such as the systematic observation across place and time.

### Contextualizing use

**Figure 2:**
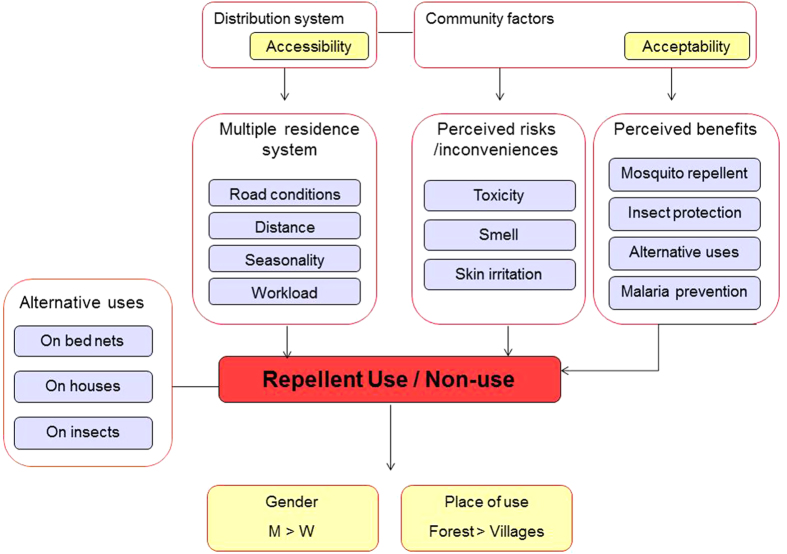
Explanatory model of repellent use showing factors contributing to the use and non-use of repellents in the study setting.

The context in which this study took place was characterized by a complex interplay of humans and mosquitoes^9^: the local population travelled between different residences located in villages, at farms and/or on rice fields, each of which located in areas of higher- or lower malaria transmission. Repellent use depended on a combination and convergence of location, time (i.e. seasonality and economic and livelihood activities), level of insect nuisance, age and gender (see Fig. 2). In order to reflect the existing behavioural heterogeneity, the concept of ‘use’ was operationalized to include social and cultural factors driving the uptake of malaria preventive measures, rather than reducing it to individual behavioural determinants alone as is often implicit in the term “compliance”^42^,^43^.

A strong preference for frequent application of the repellent was noted when performing economic and subsistence activities in the forest, mostly by men, and in places where insect nuisance is high. As insect nuisance has been shown to be one of the main stimulants for repellent use also in other contexts^44^,^45^,^46^, repellents may prove to be a effective tool for personal protection in the male deep-forest subgroup more so then for families residing at farms, despite their increased epidemiological risk^32^,^47^,^48^. Moreover, our results show that repellent use was not driven by perceived malaria risk.

Use of repellents in specific risk groups may be optimized by formative research^49^,^50^ prior and during the intervention of which kind of formula of repellent is preferred (sprays, lotions, different smells); through which channels they should be distributed; which form of community participation and level of ownership fits the local context^51^; offering an alternative and/or additional strategies to information and education campaigns.

## Limitations

No post-trial assessment on repellent use was conducted as the trial was not able to reduce residual malaria transmission at community level. While the strength of the study lies in the triangulation of qualitative and quantitative methods, including respondent dependent and independent data collection, limitations were the limited detail of the ethnographic strand on specific locations as information was expected on all study clusters due to the requirements of the large-scale epidemiological trial. The Khmer ethnicity of some of the interviewers and working within a trial context may have impacted on the data quality. Lastly, the direct observation of use was a strength in this study but complete non-reactivity is impossible and we are aware of the limitations in terms of social desirability bias.

## Conclusion

While the large-scale distribution of topical repellents may not result in a community protective effect, engaging specific risk groups, potentially contributing disproportionately to malaria transmission, could be more effective. It is, however, questionable that a further substantial reduction in malaria can be achieved by introducing standardized preventive measures without researching how these strategies impact at the local level and without optimizing community involvement strategies and/or targeting specific subgroups. Especially malaria elimination strategies for settings characterized by large percentages of asymptomatic infections^32^, such as those moving from high to low endemicity in a short period of time, with decreasing perceived benefits of preventive measures, need to be reconsidered.

## Additional Information

**How to cite this article**: Gryseels, C. *et al.* Factors influencing the use of topical repellents: implications for the effectiveness of malaria elimination strategies. *Sci. Rep.* **5**, 16847; doi: 10.1038/srep16847 (2015).

## Figures and Tables

**Table 1:** Perceived inconveniences. risks and benefits of the repellent.

	n	%
Cross-sectional Survey (N = 393)		
Conceptualization of the repellent		
Repellent is poison		
*- I don't know*	6	1.5
*- Never used any repellent*	13	3.3
*- No*	154	39.2
*- Yes*	219	55.7
*- Missing*	1	0.3
Repellent is medicine		
*- I don't know*	3	0.8
*- Never used any repellent*	13	3.3
*- No*	141	35.9
*- Yes*	235	59.8
*- Missing*	1	0.3
Repellent is both medicine and poison	146	37.2
Perceived effectiveness of the repellent		
Mosquitos still bite after spraying repellent		
*- Always*	2	0.5
*- Never*	353	89.8
*- Sometimes*	14	3.6
*- Never used any repellent*	17	4.3
*- Missing*	7	1.8
Insects still bite after spraying repellent		
*- Always*	2	0.5
*- Never*	355	90.3
*- Sometimes*	14	3.6
*- Never used any repellent*	17	4.3
*- Missing*	5	1.3
Perceived inconveniences of the repellent		
Skin-related side effects (rash. hot skin. dry skin. etc.)	338	86.0
Flu-like symptoms	128	32.6
Headache	75	19.1
Dizziness	72	18.3
Vomiting	50	12.7
Structured observation survey (N = 431 household leaders)		
Alternative uses of the repellent by respondent or family members		
Uses repellent on insects	333	77.3
Uses repellent around body	305	70.8
Uses repellent on bed net	270	62.6
Uses repellent on clothes	248	57.5
Uses repellent on walls	229	53.1
Uses repellent in bed net	197	45.7
Uses repellent around house	131	30.4
Uses repellent on blanket	100	23.2
Uses repellent under bed	91	21.1
Uses repellent on pillow	72	16.7
Uses repellent to cool body	65	15.1
Uses repellent on hair	64	14.8
Uses repellent on grass	20	4.6
Perceived mosquito density second year vs. first year of the trial		
*- Less*	240	55.7
*- Same*	108	25.1
*- More*	75	17.4
*- Don’t know*	7	1.6
*- Missing*	1	0.2

**Table 2:** Reported repellent use.

	n	%
Cross-sectional survey (N=393)		
Acces		
Received repellent last month	381	96.9
Ran out repellent before new distribution	145	36.9
Considers amount of repellent distributed:		
*- Enough*	321	81.7
*- Not enough*	37	9.4
*- Too much*	11	2.8
*- Never used any repellent*	18	4.6
*- Missing*	6	1.5
Reported use		
Always use the repellent	247	62.8
Use the repellent 7 days a week	232	59.0
Used the repellent yesterday evening	288	73.3
Used the repellent this morning	183	46.6
All of the above	134	34.1
Reported use per location		
Always use repellent in the evening when in village (N = 361)†	204	56.5
Always use repellent in the evening when at farm (N = 348)†	190	54.6
Always use repellent in the evening when at rice field (N = 155)†	79	51.0
Always use repellent in the evening when in deep forest (N = 249)†	180	72.3
Structured observation survey (N=1495 individuals in 431 households)		
Reported use per residence		
Use repellent 7 days a week when in village (N = 1315)†	622	47.3
Use repellent 7 days a week when at forest farm (N = 1325)†	719	54.3
Use repellent 7 days a week when at rice field (N = 729)†	359	49.2
Structured observation survey (N = 431 household leaders)		
Reported repellent use in the deep forest		
*- Never go to the deep forest*	112	26.0
*- Always use the repellent in the deep forest*	237	55.0
*- Sometimes use the repellent in the deep forest*	49	11.4
*- Never use the repellent in the deep forest*	25	5.8
*- Don’t have repellents*	8	1.9
Median application times per day in the forest	3	

^†^N excludes respondents that report to never stay at that location.

**Table 3:** Reported repellent use Malariometric surveys 2 and 4.

	n	%
Malariometric Survey 2 in Year 2 (N = 2490 intervention arm)		
Used the repellent yesterday	1786	71.7
Used the repellent in the last week	2056	82.5
Malariometric Survey 4 in Year 2 (N = 2730 intervention arm)		
Used the repellent yesterday	1885	69.0
Used the repellent in the last week	2230	81.7

**Table 4:** Structured Observation Survey: observed repellent use (N = 1495 individuals in 431 households).

	n	%
Observed repellent use	118	7.9
Observed repellent use per subgroup location		
Village (N = 691)†	49	7.1
Farm (N = 577)†	34	5.9
Field (N = 227)^†^	35	15.4
Observed repellent use per subgroup age category*		
Spray users (age 11+) (N = 943)	55	5.8
Lotion users (age 2–10) (N = 552)	63	11.4

^*^p < 0.05.

^†^N refers to the total amount of respondents interviewed at that location.

**Table 5:** Structured Observation Survey: reported versus observed use.

	n (%)	n (%)
	Use was observed	Use was not observed
Village		
Reported to use 7 days a week (N = 316)	32 (10.1%)	284 (89.9%)
Reported to not use or use <7 days a week (N = 375)	17 (4.5%)	358 (95.5%)
Farms		
Reported to use 7 days a week (N = 330)	26 (7.9%)	304 (92.1%)
Reported to not use or use <7 days a week (N = 247)	8 (3.2%)	239 (96.8%)
Rice fields		
Reported to use 7 days a week (N = 137)	25 (18.2%)	112 (81.8%)
Reported to not use or use <7 days a week (N = 90)	10 (11.1%)	80 (88.8%)

**Table 6:** Reasons for (non-) repellent use and in relation to other preventive measures.

	n	%
Cross-sectional survey (*N*=395)		
Reasons for using repellent		
Use repellents to protect from insect bites	295	75.1
Use repellent to protect from malaria	113	28.8
Someone in the household has had malaria before	326	83.0
Structured observation survey (N = 1495 individuals in 431 households)		
Reason for not using the repellent the evening of the visit (N = 1377)†		
*- use repellent later in evening*	717	52.1
*- run out of repellent*	178	12.9
*- forgot*	141	10.2
*- don’t like because of side effects*	77	5.6
*- don’t like because of smell*	69	5.0
*- other*	43	3.1
*- never received any repellent*	29	2.1
*- repellent not yet distributed*	27	2.0
*- forgot bottle somewhere*	25	1.8
*- did not have time*	24	1.7
*- not interested in repellent*	23	1.7
*- no mosquitos now*	22	1.6
*- too hot*	1	0.1
*- missing*	1	0.1
Do you use it sometimes or never?		
*- sometimes*	1200	87.1
*- never*	177	12.9
Structured observation survey (N = 431 household leaders)		
Repellent use in relation to other preventive measures:		
No longer use repellent when using smoke from fire		
*- Yes*	49	11.4
*- No*	292	67.7
*- Never make fire*	55	12.8
*- Never use repellent*	35	8.1
No longer use repellent when using smoke from cigarettes		
*- Yes*	59	13.7
*- No*	216	50.1
*- Never smoke cigarettes*	121	28.1
*- Never use repellent*	35	8.1
Use bed net less often when using repellents		
*- Yes*	60	13.9
*- No*	336	78.0
*- Never use bed nets*	7	1.6
*- Never use repellent*	27	6.3
*- Missing*	1	0.2
Someone in the household has had malaria in the last year	164	45.4

^†^N refers only to those who had not used the repellent on the evening of the interview.

**Table 7:** Structured observation survey: reported insect nuisance related to repellent use (N = 431 household leaders).

	n	%
Most mosquito nuisance during:		
* - dry season*	26	6.0
* - rainy season*	384	89.1
* - same*	21	4.9
Most insect nuisance during:		
* - dry season*	24	5.6
* - rainy season*	388	90.0
* - same*	19	4.4
Most mosquito nuisance in (multiple options possible):		
* *Deep forest	143	33.2
* *Forest around farm	134	31.1
* *Forest around village	37	8.6
* *Bamboo forest	34	7.9
* *Farm	47	10.9
* *Field	14	3.2
* *Village	20	4.6
